# How companions speak on patients’ behalf without undermining their autonomy: Findings from a conversation analytic study of palliative care consultations

**DOI:** 10.1111/1467-9566.13427

**Published:** 2022-02-14

**Authors:** Marco Pino, Victoria Land

**Affiliations:** ^1^ School of Social Sciences and Humanities Loughborough University Loughborough UK

**Keywords:** companions, conversation analysis, cues, end of life, gaze, hospice, offers, palliative care, questions

## Abstract

Companions are individuals who support patients and attend health‐care appointments with them. Several studies characterised companions’ participation in broad terms, glossing over the details of how they time and design their actions, and how patients and health‐care practitioners (HCPs) respond to them. This article aims to examine these aspects in detail by using conversation analysis, focusing on actions whereby companions speak on patients’ behalf—mentioning delicate aspects of patients’ experience (specifically, by alluding to patients’ thoughts or feelings about dying). Some studies suggested that these actions undermine patients’ autonomy. By contrast, through examination of palliative care consultations in a UK hospice, we found that these interventions are warranted by contextual circumstances: they are either invited by patients or HCPs (through questions or gaze) or volunteered to help with the progression of an activity (e.g. when a patient does not answer an HCP’s question). Additionally, all parties collaborate in constructing these companion interventions as temporary departures from an otherwise prevailing normative orientation to patients’ right to speak for themselves. The study contributes to the sociology of health and illness by characterising how companions contribute to the ways in which participants coordinate their relative rights and responsibilities, and ultimately their relationships, within health‐care interactions.

## INTRODUCTION

An important task for the sociology of health and illness is to examine the practical problems that health‐care providers and users face; how they tackle them; and how, in the process of doing so, they coordinate their relative rights and responsibilities, thus shaping the structure of their relationships. This is often discussed in the context of doctor–patient relationships and interactions (Heritage & Maynard, 2011; May, 2011; Weiss & Lonnquist, 2015), thus overlooking the involvement of other key players, including companions—people who support patients and attend health‐care appointments with them. Companions’ participation is sometimes discussed as a complicating factor—‘[t]he addition of another person who has questions, needs information, and may distract from conversation with the patient’ (Pecchioni, 2014, p. 1044). These perspectives implicitly treat companions’ participation as accessory. This is especially problematic for settings where companions are likely to play a significant part in the care and support of patients, such as when patients are older, frailer and manage life‐limiting, progressive illnesses (Laidsaar‐Powell et al., 2013; Wolff & Roter, 2011). This includes palliative care, the context of our study. This article addresses the need for observational research that examines the ways in which companions’ actions contribute to health‐care interactions involving patients and health‐care practitioners (HCPs). We do so by examining previously undocumented actions whereby companions share sensitive aspects of patients’ experience on their behalf.

### Research on companions’ participation

Companion participation has been examined in several adult health‐care settings (Clayman et al., 2005; Eggly et al., 2006, 2011; Fioramonte & Vasquez, 2019; Hasselkus, 1992; Ishikawa et al., 2005; Karnieli‐Miller et al., 2012; Mazer et al., 2014; Street & Gordon, 2008; Wolff et al., 2017). Some studies conceptualised companion participation in terms of the *roles* they play relative to other participants, particularly patients. These roles can be broadly grouped into: (a) a passive (Adelman et al., 1987) or observer role (Ishikawa et al., 2005; Street & Gordon, 2008), involving witnessing the patient's interactions with HCPs without intervening; (b) an unsupportive or detrimental role, such as acting antagonistically towards the patient (Adelman et al., 1987; Ishikawa et al., 2005), hindering or undermining their actions; and (c) a helpful or supportive role including facilitating the patient's actions and advocating for them (Adelman et al., 1987; Ishikawa et al., 2005; Street & Gordon, 2008).

Although roles intuitively offer useful heuristics to understand companion participation, they are very broad characterisations, each lumping together very different forms of participation. For example, the supportive role would be embodied in diverse actions such as when companions ask the doctor a question on behalf of the patient or when they prompt the patient to ask a question (Ishikawa et al., 2005). Crucially, these groupings are based on generalisations about the functions and effects of certain actions, regardless of how they are prompted and realised on particular occasions and of how patients and HCPs respond to them. Additional problems are associated with the procedures used to identify companions’ actions and evaluate their effects. We discuss this in the next section.

### Speaking on behalf of patients

Our study focuses on actions whereby companions mention sensitive aspects of patients’ experience on their behalf (see Mazer et al., 2014). Some actions whereby companions speak on behalf of patients have previously been considered as autonomy‐enhancing, such as when companions clarify aspects of patients’ medical history (Clayman et al., 2005; Ishikawa et al., 2005; Wolff et al., 2017). However, one of the ways of speaking on behalf of patients—answering a question that an HCP addressed to the patient—has been considered as autonomy‐detracting (Clayman et al., 2005). An exception to this view is Hasselkus’s (1992) study of geriatric consultations, in which answering on behalf of a patient is seen as a form of mediation.

Shared across these studies is the use of a priori criteria to evaluate classes of companion actions as positive or negative regardless of how they are realised and responded to on particular occasions. Compounding the limitations of this approach is a lack of analytic detail necessary to understand what may prompt (and possibly warrant) companions’ interventions done on behalf of patients. For instance, there is a difference between answering a question on behalf of an addressed recipient before they have an opportunity to respond, versus doing so after a silence has emerged (in which the addressed recipient has had a first opportunity to respond but has not done so; Stivers & Robinson, 2006), versus doing so after the addressed recipient has recruited another participant to respond on their behalf, for example by gazing at them (Tiitinen & Ruusuvuori, 2012). The aforementioned studies did not make these distinctions. Published transcripts did not include a representation of silences in interactions (Clayman et al., 2005; Mazer et al., 2014; Wolff et al., 2017), thus omitting information on what may sometimes prompt companions to respond on patients’ behalf (by contrast, see Fioramonte & Vasquez [2019, p. 140, Extract 3.3, line 158] for an example where a companion answers on a patient's behalf after a silence emerges following an HCP’s question, in which the patient does not respond). Additionally, those analyses did not reportedly take into account visible conduct including gaze—thus overlooking a key form of recruitment that can prompt (and warrant) companions’ responses on patients’ behalf (additionally, this visual level of analysis was entirely precluded in studies that relied on audio recordings [Mazer et al., 2014; Wolff et al., 2017]). Our point is that companions’ interventions on behalf of patients should not be treated as autonomy‐detracting before establishing whether other participants’ actions or other local contingencies prompted (and warranted) those interventions.

In this article, we use conversation analysis (CA; Sidnell & Stivers, 2013), which involves detailed examination of recorded interpersonal interactions, focusing on the resources (including language and bodily conduct) that participants use to accomplish social actions. Central to CA is attention to the ways in which the functions and effects of actions are shaped by their design and positioning, and to the ways in which participants display understandings of one another's actions and their effects through the ways in which they respond to them. An example that is relevant to our study is Antaki and Chinn’s (2019) CA‐based examination of companion participation in primary care consultations involving patients with intellectual disabilities. The authors focused on cases where companions answer questions directed at the patient. The authors distinguished between (a) companion interventions that are prompted (and warranted) by specific contingencies in the interaction and are concurrently designed in ways that support (rather than undermine) patients’ prerogative to speak from themselves (and thus their autonomy); and (b) companion interventions that are not thus prompted and therefore emerge as more intrusive and detracting from patients’ autonomy. That study shows how the CA approach, focusing on the situated contingencies that prompt participants’ actions, can offer a more nuanced picture of companion participation compared to approaches that conceptualise it in terms of summative roles or evaluate the impact of companions’ actions as positive or negative based solely upon exogenous criteria.

### Mentioning delicate aspects of patients’ experience in palliative care interactions

Our study is part of a research programme whose aims include identifying ways in which patients’ end of life (EoL) is discussed in hospice‐based palliative care interactions. Palliative care adopts a holistic approach to supporting patients whose disease does not respond to curative treatment, thus extending the focus beyond the biomedical to embrace psychological, social and spiritual matters (Faull, 2015). Working on recorded interactions involving hospice HCPs, patients and companions, we observed that sometimes companions mention delicate aspects of patients’ experience, which possibly relate to patients’ own thoughts or emotions associated with the prospect of dying. We call these *EoL*‐*implicative* aspects of patients’ experience. It struck us as peculiar that companions would mention these on behalf of patients.

Sacks (1984) proposed that people treat personal experience as a special domain of knowledge: those who own the experience are ordinarily treated by others as holding the right to articulate it—as opposed to having it articulated by others on their behalf (see also Lerner, 1996). In a study of counselling sessions involving HIV‐positive patients and their companions in the 1980s, Peräkylä and Silverman (1991) observed that the participants structured their interactions in ways that regularly reaffirmed the patient's right to have the final say about their personal experiences. Therefore, we wondered what might prompt companions to share delicate aspects of patients’ experience and what they accomplish by doing so.

Our aim is to investigate what prompts actions whereby companions mention EoL‐implicative aspects of patients’ experience on their behalf; how companions design them; and how patients and HCPs respond. In those moments, companions apparently trespass into patients’ domain of personal experience and concurrently manage (and thus make visible) the boundary between their own prerogatives and those of patients—boundaries that otherwise remain invisible in so far as participants usually respect them tacitly. Examining cases where companions mention EoL‐implicative aspects of patients’ experience is therefore an opportunity to observe how participants manage their relative rights and responsibilities in real time, and how they construct their relationships through the ways in which they accomplish delicate tasks in their interactions. This has implications for understanding how patient autonomy can be hindered or supported at the ground level of everyday interactions within health‐care settings.

## METHODS

### Data

We recorded health‐care outpatient and inpatient consultations in a large UK hospice, with ethical approval obtained from UK NRES Committees Coventry & Warwickshire (Ref: 14/WM/0128) in 2014, and Nottingham 2 (Ref: 17/EM/0037) in 2017.

All interactions were in spoken English. Participants gave consent for the use of pseudonymised transcripts in publications. Patients had been diagnosed with life‐limiting (sometimes called ‘terminal’) conditions and were attending the hospice for review or management of difficult symptoms (physical or emotional) and/or help with planning future care. The hospice is an independent charitable organisation much of whose services are provided to the UK National Health Service, and for which patients and their companions are not charged. The dataset comprises 85 consultations (72 audiovisually recorded, 13 audio‐recorded) involving 85 patients, 38 companions, six palliative medicine doctors, three physiotherapists and five occupational therapists. All examples in this article are from videorecorded interactions.

### Analysis

For this article, we collected instances where companions mention *EoL*‐*implicative aspects* of patients’ experience; that is, they mention something that can be heard as related to the patient's prospect of dying. Because they are sometimes ambiguous or allusive, initial mentions of EoL‐implicative aspects have been conceptualised as ‘cues’ in previous studies, that is ‘indirect hint[s] of an underlying feeling’ (Zimmermann et al., 2007, p. 438). Indeed, we observed that participants in our data sometimes treat them as being related to the patient's EoL in subsequent interaction, and sometimes they do not. When they do, this can then lead to talk that focuses on the patient's thoughts or feelings about dying. In this article, we adopt an understanding of ‘cue’ that eschews the mentalist assumption that companions might produce them out of the need or intention to discuss the patient's EoL. We simply observe that those descriptions can be (although they need not be) the starting point for trajectories that lead to talk about the patient's EoL.

Our approach to identifying candidate cues was informed by CA methods. We initially collected cases in which participants observably treat a companion's turn as EoL‐implicative (we exemplify this form of internal evidence in the analysis of Extract 1a, 1b). We then extended our collection to cases where companions produce similar kinds of turn (based on sequential context, focal action and type of concern raised in the turn) but are not treated as EoL implicative on those particular occasions (see Schegloff, 1996, endnote 8).

We identified 12 instances where companions mention EoL‐implicative aspects of patients’ experience. This happens in contexts where companions respond to HCP sequence‐initiating actions (Schegloff, 2007) that select the patient to respond: either a question (see examples 1, 2, 3 and 5) or the offer of a health‐care measure or service (see examples 4 and 6). We did not use specialist software to analyse the data but compiled our analytic notes in a separate document for each case. We then used a spreadsheet to collate and compare features across cases (including the sequential context of companions’ actions, their design and other participants’ responses). We transcribed target sequences using notation that is conventional in CA to represent participants’ talk (Jefferson, 2004) and visible conduct (Mondada, 2018). In this article, we keep representations of visible conduct to a minimum in order to maximise accessibility of our transcripts.

## RESULTS

### Mentioning EoL‐implicative aspects of patients’ experience

Before our main analysis, we first demonstrate that a companion's mention of an aspect of the patient's experience (panic in this case) can work interactionally as an EoL‐implicative cue by initiating a trajectory leading to talk about the patient's EoL. Eashan, a patient with neck cancer, and his brother Rajesh are attending an outpatient consultation with a doctor. Before Extract 1a, 1b, they have talked about Eashan's persistent cough. The doctor then changed topic, asking Eashan whether he is taking blood‐thinning medication (data not shown). As Extract 1a begins, Rajesh goes back to the topic of cough.

EXTRACT 1a **VERDISDOC_31 14.35 VT310 EL_DOC31.2 MP ‘He panics’**

P‐Eas = Eashan (patient); C‐Raj = Rajesh (companion); Doc = doctor (Mick).

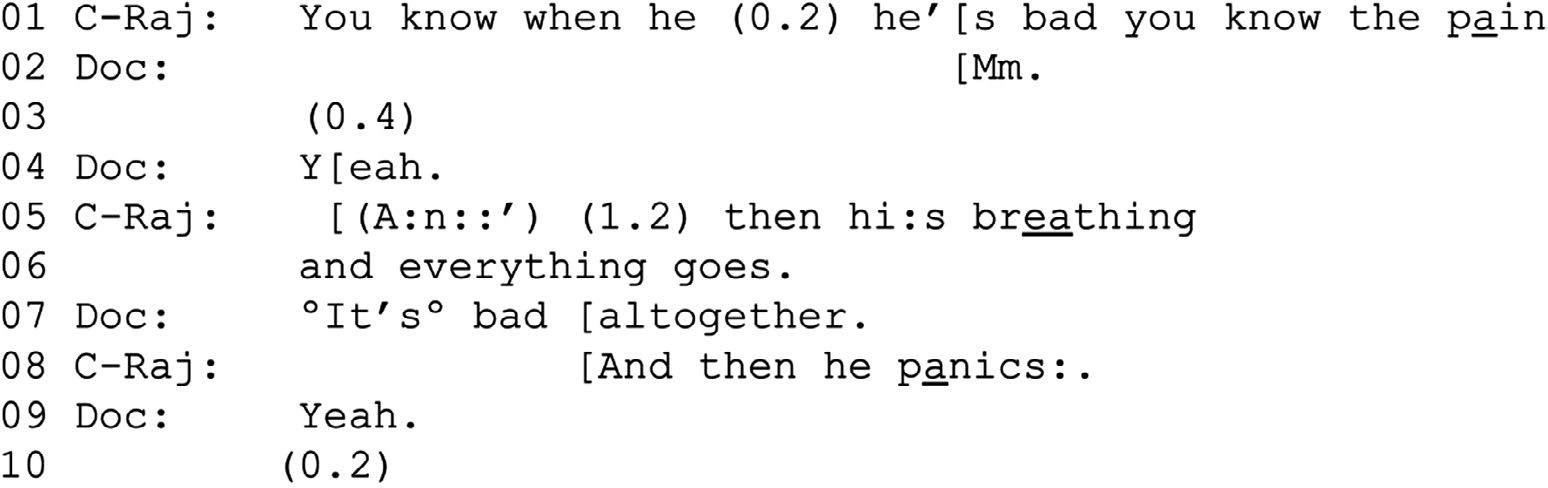

Addressing the doctor, Rajesh describes times when Eashan is in pain, experiences breathlessness and panics (lines 1, 5–6 and 8). Participants in our data recurrently treat references to panic during episodes of breathlessness as EoL‐implicative by elaborating them in terms of fear of dying. This can subsequently lead to further talk about the patient's EoL. Extract 1b shows this development. After 20 s where the participants talk about Eashan's cough (data not shown), the doctor asks Eashan about the panic Rajesh mentioned.

EXTRACT 1b **(20 s after 1a)**

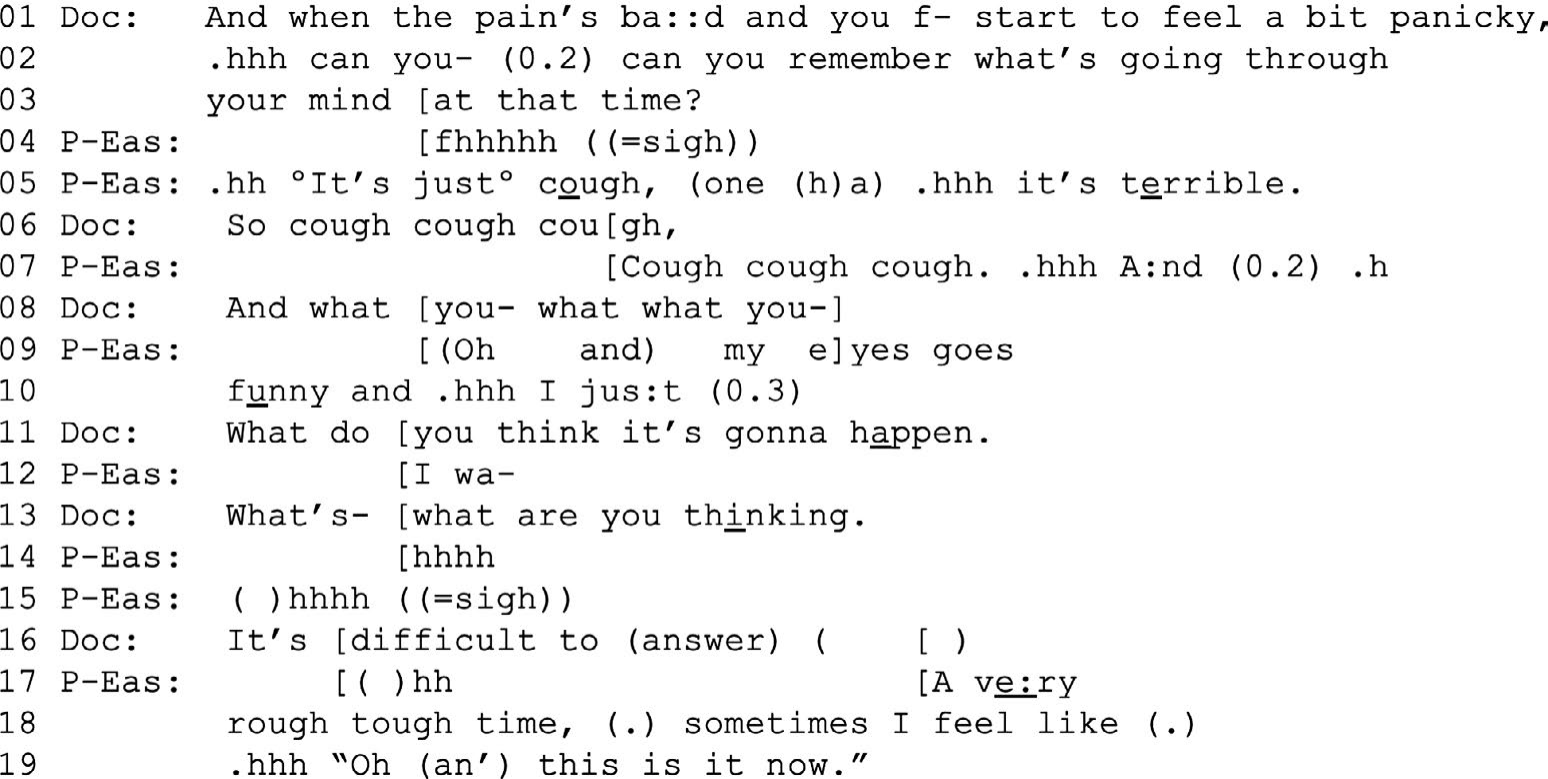

The doctor invites Eashan to share ‘what is going through his mind’ when he panics (lines 1–3). Eashan initially responds that it is ‘just cough’ (line 5), but after the doctor pursues an alternative answer, increasingly alluding to concerns about the future (lines 6, 8, 11, 13 and 16), Eashan shares that, in those moments, he fears that he might die (with the idiomatic ‘this is it now’, lines 17–19). In subsequent interaction, not shown here, the participants further talk about Eashan's fear of dying.

Extract 1a, 1b shows how a companion's mention of an aspect of the patient's experience (panic) works interactionally as an EoL‐implicative cue, initiating a trajectory leading to talk about the patient's EoL. The EoL implicativeness of some descriptions is linked to the setting in which these interactions take place. In Extract 1a, 1b, the participants already know about Eashan's deteriorating condition, and in this context, a reference to panic during episodes of breathlessness can be heard as implying fear of dying. Additionally, talk about patients’ EoL is relevant in the hospice setting because promoting it is part of the hospice clinical staff's remit (on the omni‐relevance of category‐bound considerations within a setting; see Sacks, 1992, Spring 1966, Lecture 6).

In the rest of this article, we examine how companions mention EoL‐implicative aspects of patients’ experience, focusing on what prompts them and how they design them. We will show that companions do so in circumstances that warrant a departure from an otherwise prevailing orientation to patients’ right to speak for themselves.

### The companion is invited to intervene

In these cases, the patient or an HCP promotes the companion's intervention with a question or through gaze.

#### The HCP asks the companion a question

In the next example, Lewis, a patient with metastatic cancer, and his partner Arthur are attending an outpatient appointment with a doctor. Lewis has been drowsy throughout the consultation, and Arthur has done quite a bit of talking on his behalf (data not shown). In Extract 2, the doctor starts to move towards a possible closure of the consultation by asking Lewis whether he has anything else to ‘mention’ (line 1). After Lewis answers negatively (lines 5 and 9), the doctor directs the same question at Arthur (line 11). In the following transcripts, we highlight in grey the action or event that warrants the companion's intervention on the patient's behalf.

EXTRACT 2 **VERDISDOC_20 18,55 VT553 EL_DOC20.1 VL ‘Very depressed’**

P‐Lew = Lewis (patient); C‐Art = Arthur (companion); Doc = doctor Mick.

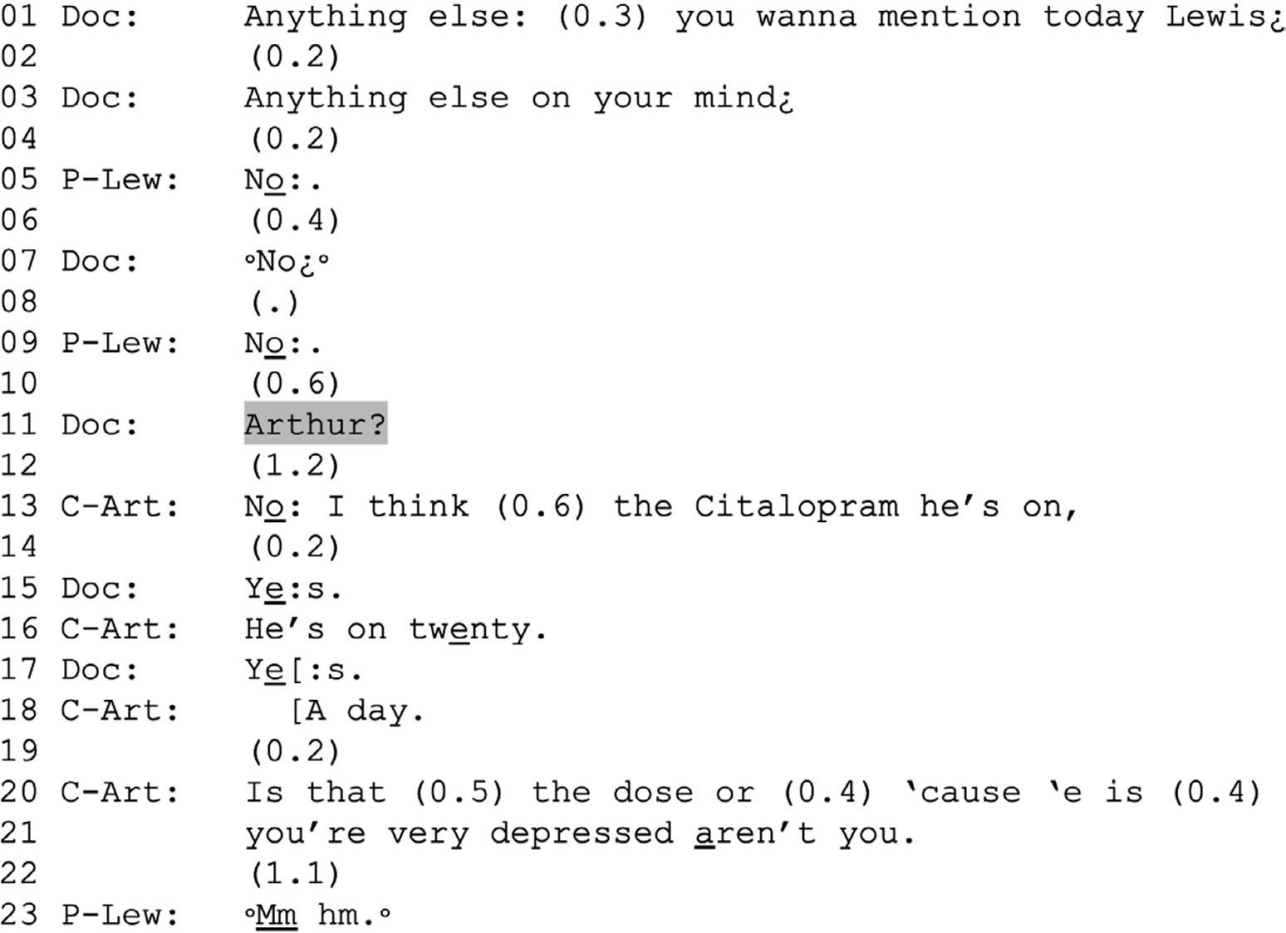

By addressing his question to Lewis first, the doctor implicitly treats him as having the right to be the first to raise concerns in the consultation. Arthur supports this by not intervening to rectify or add to Lewis's negative answer at points where he could do so (lines 6 and 10). That he *could* do so is evidenced by the fact that, as it emerges from line 13, Lewis has not raised something that Arthur treats as important: that the current dose of his anti‐depressant might not be enough (lines 13, 16, 18 and 20–21). The doctor's question addressed to Arthur (line 11) gives him a warrant for raising this (see Doehring, 2019, pp. 42–50).

Arthur treats raising this matter as delicate in so far as it entails contradicting Lewis’s own answer and because it falls into Lewis's domain of experience. First, after a substantive delay (line 12) Arthur confirms Lewis's answer (‘No’, line 13; see Sacks, 1987, p. 62; Schegloff, 2007, pp. 66–67) before departing from it by mentioning the anti‐depressant. Second, after introducing the anti‐depressant (line 13) and its dose (lines 16 and 18), Arthur starts to ask the doctor whether that dose is sufficient (line 20), thereby implying (rather than stating) a problem with it, but then suspends production of the question to address Lewis (line 21), proposing that he is depressed. This accounts for the projected request to increase the anti‐depressant and is also a statement about something that Lewis is more knowledgeable of, making relevant for him to confirm or disconfirm (Pomerantz, 1980); Arthur further seeks Lewis's confirmation through the tag question ‘aren't you’ (line 21). Participants in our data recurrently treat references to depression and low mood as EoL‐implicative; HCPs recurrently invite patients to say more (the doctor in Extract 2 will do so in subsequent interaction, not shown here), and this can lead to talk about the patient's EoL.

Extract 2 exemplifies how participants manage their relative rights and responsibilities in our data. By addressing his question to both Lewis and Arthur, the doctor treats them both as being entitled to raise questions and concerns, but not in the same way. By addressing Lewis first, the doctor treats him as holding primary rights to raise matters. Arthur supports this through the positioning and design of his intervention, embodying deference to Lewis as the most authoritative source on matters pertaining to him (Lerner, 1992, p. 252). Additionally, through Arthur's actions Lewis is granted the right to have the final say on those matters (Peräkylä & Silverman, 1991). Arthur thereby treats speaking on Lewis's behalf as an accountable matter—a departure from an otherwise prevailing orientation to Lewis's right to speak for himself (Lerner, 1996).

#### The HCP gazes at the companion

Lee, a patient with motor neurone disease (a degenerative neurological condition also known as amyotrophic lateral sclerosis), and his wife Sharon are attending an outpatient appointment with a doctor. The doctor has asked about Lee's breathing difficulties, a consequence of his condition (data not shown). As Extract 3 begins, the doctor asks about the position in which Lee lies at night (flat or propped up; lines 1–2 and 4). Lee responds that he is usually propped up, but that position prevents him from sleeping, so he ends up trying to lie down (lines 5–7 and 9). Participants’ gaze direction is shown in the transcript using Mondada’s (2018) notation.

EXTRACT 3 **VERDISDOC_06.1 10,03 VT 363 EL_DOC06.1 MP ‘He has this waking up panicking’**

P‐Lee = Lee (patient); C‐Sha = Sharon (companion); Doc = doctor Mick.

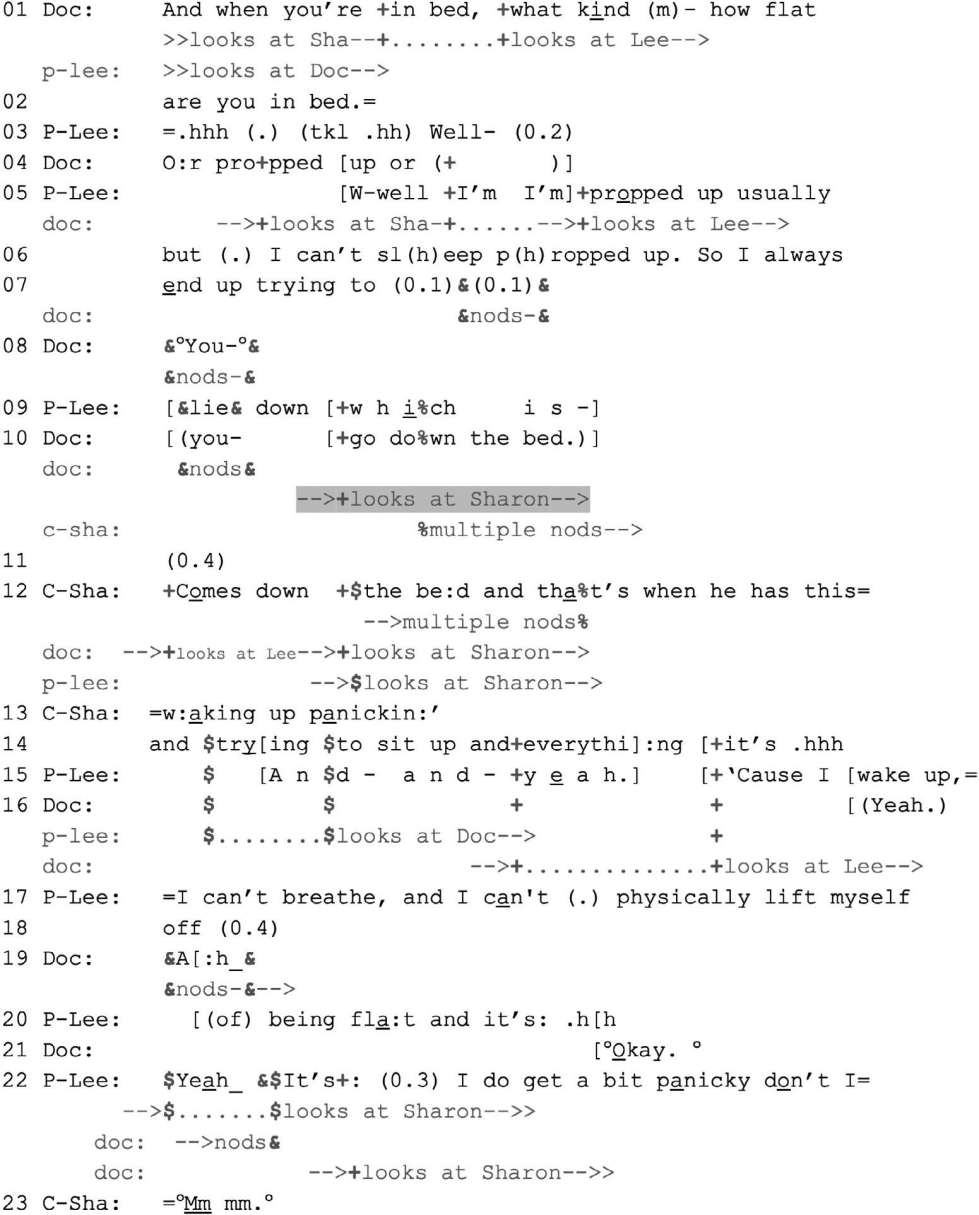

The participants collaboratively maintain a distribution of prerogatives that grants Lee primary rights to voice his own experience. The doctor addresses his question (lines 1–2) to Lee. When Lee hearably hesitates at line 3, the doctor starts to revise the presuppositions embodied in his question (now asking if Lee sleeps propped up rather than flat, line 4) and in doing so looks at Sharon, thereby treating her as someone who might also have knowledge about those matters and assist with answering. She nevertheless does not intervene here, thereby tacitly treating Lee as holding the right to be the first to answer.

After Lee starts to answer (lines 5–7), the doctor formulates the upshot of that answer (that Lee ‘goes down the bed’, line 10; Heritage & Watson, 1979). He addresses Lee (‘you’ in line 10 being said whilst gazing at Lee), thus treating him as having the primary right to confirm or disconfirm. The doctor simultaneously turns towards Sharon (upon pronouncing ‘go’ in line 10), thus tacitly treating her as *also* knowledgeable about this matter. This time, Sharon immediately starts to nod (midway through the doctor's next word, ‘down’, line 10). Following a 0.4‐second silence (line 11), she confirms the doctor's formulation (‘comes down the bed’, line 12) and then expands by reporting that Lee wakes up panicking (lines 12–13). By stepping in at this point, Sharon ‘goes second’, after Lee has had an opportunity to be the first to answer the doctor's question. Sharon acts as a ‘story consociate’ whose ‘limited entry’ into her husband's report‐in‐progress is made relevant by a recipient's action (here, the doctor's gaze; Lerner, 1992, pp. 260–261). Sharon's reference to panic is a statement about Lee's experience, making relevant for him to confirm or disconfirm (Pomerantz, 1980). He confirms and expands (lines 15, 17–18, 20 and 22), thereby showing an orientation to his right to have a say on those matters (Peräkylä & Silverman, 1991). As Extract 1a, 1b exemplified, reports of panic associated with breathlessness are recurrently treated as EoL‐implicative in our data.

#### The patient gazes at the companion

Tim, a patient with a condition causing fibrosis of the lungs, and his wife Bev are attending an outpatient consultation with an occupational therapist (OT). At the start of Extract 4, the OT is showing Tim a rollator—a four‐wheeled walking aid that could help him conserve his breathing. As the OT describes the rollator (lines 1, 4 and 7–8), Tim remains silent. By contrast, Bev treats the OT’s description as newsworthy (line 3) and volunteers the description of a positive feature (lines 5–6), possibly implying a favourable position towards the rollator. The OT brings her description, which implicitly conveys an offer, to a close with a summary (line 10). She keeps gazing at Tim in the ensuing silence, ostensibly waiting for him to take a position. After considerable silence, interspersed with Tim's and the OT’s quietly uttered and suspended turn starts (lines 12–15), Tim smiles and gazes at Bev (line 16).

EXTRACT 4 **VERDIS_AHP28 VT836 25.00 EL 28.1 ‘He'll hang on until the end’**

P‐Tim = Tim (patient); C‐Bev = Bev (companion); OT = occupational therapist Gina. Another occupational therapist is present (Julianne).

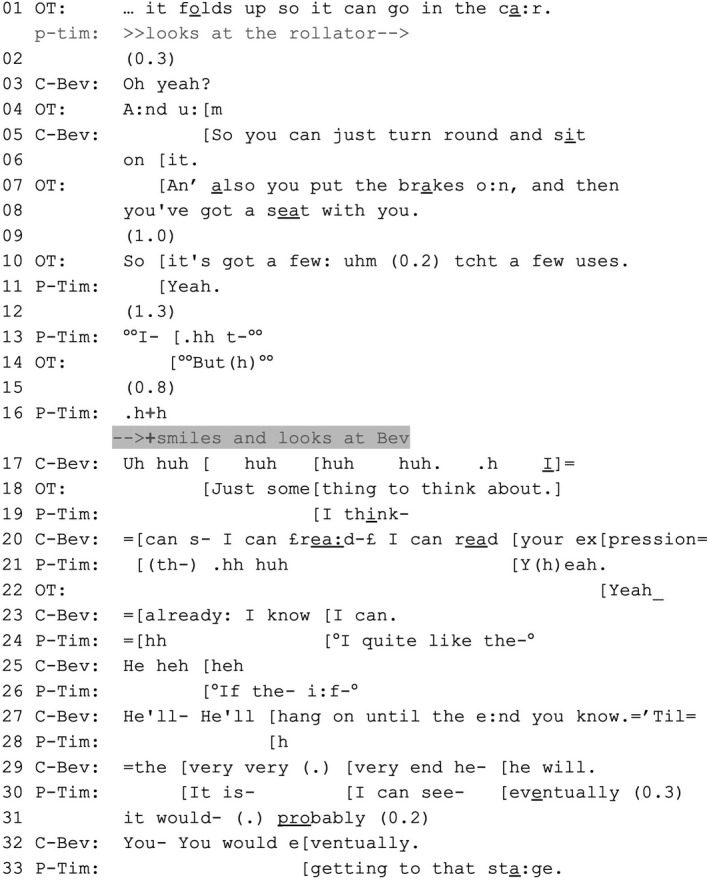

Before Tim looks at her (line 16), Bev does not intervene, thereby deferring to Tim's right to take a position on the rollator. However, when Tim looks at Bev, she immediately starts to laugh (line 17). In overlap, the OT treats Tim's embodied conduct (the prolonged silence followed by his smile and gaze towards Bev) as a sign of reluctance towards the walking aid (Pomerantz, 1984); she reframes the matter as ‘just something to think about’ (line 18). Meanwhile, despite Tim starting to take a position (with the overlapping and cut‐off ‘I think’ in line 19), Bev builds towards her own response. After laughing, she claims to be able to ‘read’ Tim's expression (lines 17, 20 and 23). Tim again tries to voice a position on the rollator (line 24); Bev disattends this with her laughter in line 25 and then articulates Tim's position on his behalf whilst addressing the OT (lines 27 and 29). She does so in a way that is hearably EoL‐implicative, articulating Tim's position towards his illness progression as an account for his reluctance to use a walking aid (‘He'll hang on until the end’). ‘The end’ alludes to advanced physical deterioration or death, which Tim picks up on and further alludes to (‘that stage’, line 33). With her actions, Bev treats Tim's smile and gaze towards her as an invitation to intervene. She intercedes on Tim's behalf (Lerner, 2019), relieving him from the burden of delivering a projectable rejection, that is an interactionally dispreferred response to the OT’s offer.

Extract 4 differs from Extract 2 in that the companion does not frame her response tentatively and, rather, ‘upgrades’ her claim to know the patient's experience (see Peräkylä & Silverman, 1991, p. 452). But in doing so Bev still treats Tim as the owner of that experience, which she claims to be able to infer by ‘reading’ his facial expression (line 20), thereby invoking her intimate relationship with him and the knowledge that comes with it (see Rossi & Stivers, 2021). Notably, Tim states his position after Bev, thus claiming his right to have the final say on matters pertaining to him (lines 30–31 and 33).

### Other warrants for intervening on the patient's behalf

In other cases, the companion's intervention is not invited, either explicitly with a question or through gaze. The intervention is nevertheless warranted by the fact that the patient does not answer an HCP’s question, or only responds in way that can be treated as incomplete or incorrect. Here, we only exemplify the former due to space constraints.

#### The patient does not answer a question by an HCP

Patricia, a patient with motor neurone disease, and her husband Lennie are attending an outpatient consultation with a doctor and an occupational therapist. Before Extract 5, Patricia has reached for a mug, saying that her mouth is dry, and started drinking from it (data not shown). As Extract 5 begins, the doctor asks her about her breathlessness (lines 1–3). Lennie turns to Patricia midway through the doctor's question (line 2). Patricia produces one cough towards the end of the doctor's question (line 4) and then keeps drinking from the mug. A 1.1‐second silence emerges (line 5).

EXTRACT 5 **VERDISAHP29 VT533 25,06 EL29.1 MP ‘panic attack’**

P‐Pat = Patricia (patient); C‐Len = Lennie (companion); Doc = doctor Hannah. An occupational therapist (Michelle) is present.

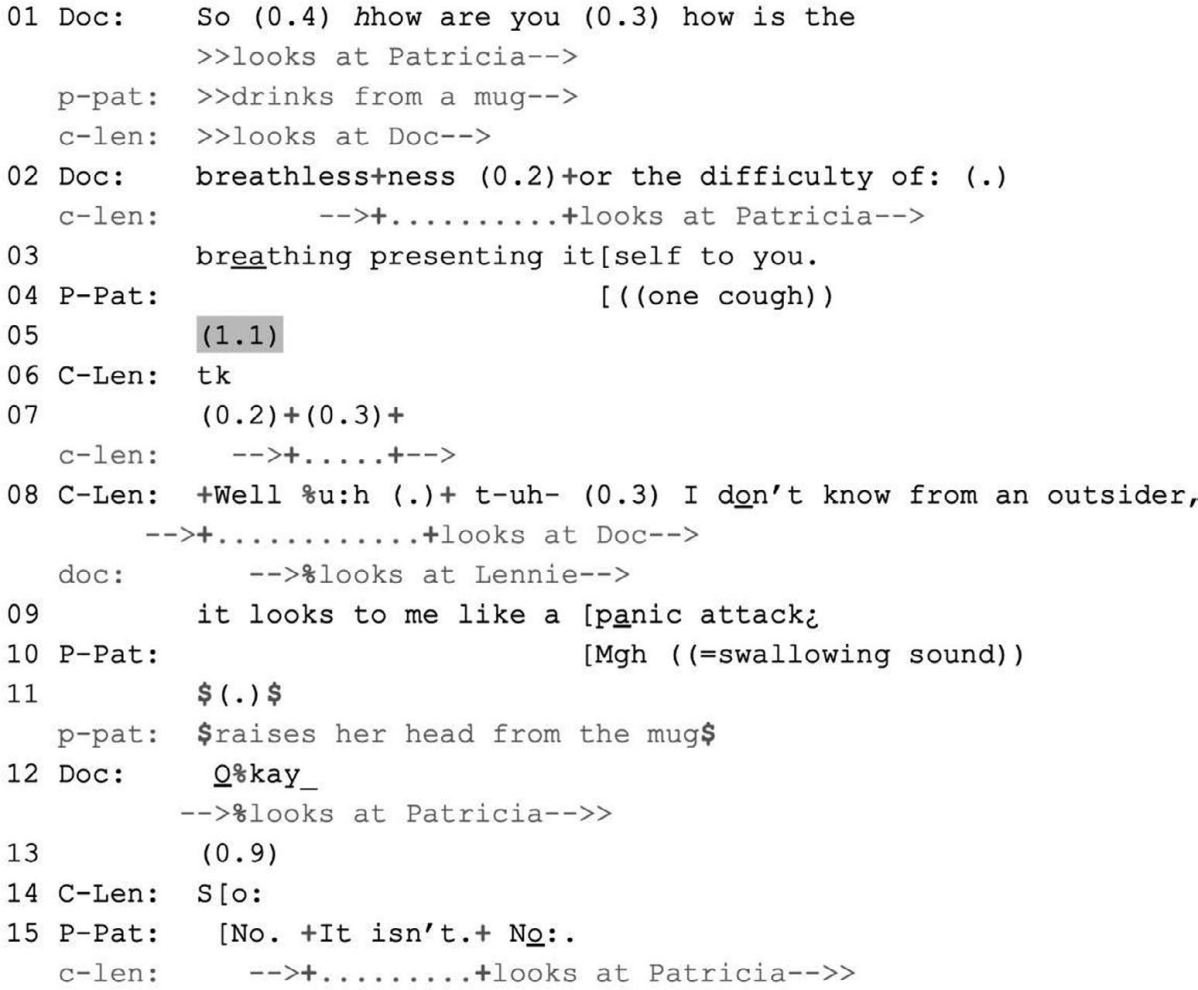

Addressed recipients of a question are expected to respond upon its possible completion, when turn transition becomes relevant (Sacks et al., 1974). However, when an addressed recipient does not answer, a non‐addressed recipient (here, Lennie) can step in and answer for them, thus prioritising forwarding the activity with which the question is concerned over waiting for the addressed recipient to respond (Stivers & Robinson, 2006).

Up to the silence in line 5, Lennie monitors Patricia and does not intervene, deferring to her right to answer. The doctor also gazes at Patricia, who nevertheless does not reciprocate (Patricia's gaze is directed at a lower point in space) and keeps drinking. Patricia's demeanour thus indicates that she is not starting to respond, and this provides Lennie with a warrant for intervening. Unlike the cases in the previous section, no‐one prompts Lennie's intervention, which is therefore volunteered (neither the doctor nor Patricia gaze at him).

Lennie hearably parts his lips (line 6) and starts to turn towards the doctor (line 7). He then answers the question on Patricia's behalf by speculating that the breathlessness causes her panic attacks (lines 8–9). He constructs his answer tentatively, downgrading his epistemic access to Patricia's inner experience—he positions himself as ‘an outsider’ whose observations are based on inference rather than first‐hand knowledge. In doing so, he mentions an EoL‐implicative aspect of Patricia's experience: that she panics during episodes of breathlessness. Midway through her ‘Okay’ (produced in receipt of Lennie's answer, line 12), the doctor turns towards Patricia (possibly prompted by Patricia's swallowing sound in line 10 and lifting of her head and gaze, which may display she is preparing to speak). In this way, the doctor displays an orientation to Patricia's right to have the final say on matters pertaining to her experience. Patricia further claims this right by disagreeing with Lennie's assessment (line 15).

### A negative case

We have seen that companions do not override patients’ right to respond. They either step in, answering a question or an offer addressed to the patient, after another participant selects them to do so (e.g. through gaze; Extract 3 and 4); or after a silence, in which the patient can be seen to have momentarily relinquished the right and obligation to respond (Extract 5). We identified only one exception, in which a companion responds on a patient's behalf before the patient has an opportunity to do so and without anyone prompting their intervention.

Bill, a patient with chronic obstructive pulmonary disease and lung fibrosis, and his wife May are attending an outpatient appointment with a physiotherapist and an OT. The two HCPs offered to show Bill breathing techniques to help manage his breathlessness. Bill said that he previously tried some techniques, which made no difference. Just before Extract 6, the physiotherapist asked Bill whether he thinks that nothing they might discuss would help him, and he confirmed that (data now shown). As Extract 6 begins, the physiotherapist validates Bill's answer (lines 1–6, 8–13 and 15–16), proposing that it helps the HCPs understand what types of help they might offer, thus further pursuing the possibility of offering services.

EXTRACT 6 **VERDISAHP_44 33,12 VT954 EL_AHP44.2 MP ‘Fed up to the teeth’**

P‐Bil = Bill (patient); C‐May = May (companion); PT = physiotherapist (Raquel); OT = occupational therapist (Gina).

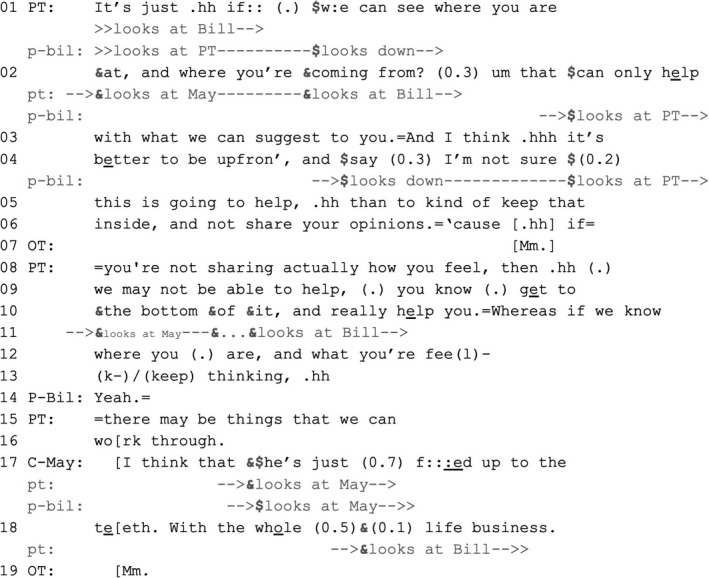

Bill's ‘yeah’ in line 14 is a continuer (Schegloff, 1982), displaying his understanding that the physiotherapist's turn has not come to a point of possible completion where a response from him will be expected (particularly as ‘if’ in line 10 projects a compound turn unit whose second component has not yet been produced by the end of line 13; Lerner, 1991). By contrast, the possible completion of the turn component at the end of line 16 would make relevant a response from Bill that addresses the physiotherapist's offer of services.

Bill's companion, May, starts to respond before possible completion of the physiotherapist's turn (in line 17, through what Jefferson [1984] termed a pre‐completor onset), thereby overriding Bill's right to respond (Antaki & Chinn, 2019). This intervention is not prompted: the physiotherapist and Bill maintain mutual gaze in lines 14–16 and do not gaze towards May. One explanation for this divergent pattern is that the HCPs have pursued Bill's cooperation with the agenda they set out for the consultation (offering advice on how to alleviate his breathing difficulties), which he has repeatedly resisted. Intervening in overlap in line 17 helps May pre‐empt a point where Bill might be in a position to deliver another dispreferred response to that offer. May accounts for Bill's reluctance to accept by invoking an EoL‐implicative aspect of his experience: that he is tired of living (lines 17–18), which Bill confirms in subsequent interaction (not shown here), sharing his thoughts about euthanasia (despite this not being legal in the UK, where this consultation was recorded). Therefore, by intervening pre‐emptively May can be seen to be interceding on behalf of Bill (Lerner, 2019). We should further note that the physiotherapist displays an orientation to Bill's right to have the final say: she turns towards him before possible completion of May's turn (line 18). Bill further claims that right by expanding in terms of his thoughts about euthanasia (data not shown). Extract 6 further shows how a companion's action can assist and qualify HCP‐patient transactions when interactional tensions arise—a matter that we further discuss in the final part of this article.

## DISCUSSION AND CONCLUSION

Our initial puzzle in this study of hospice consultations came from noticing that companions sometimes mention EoL‐implicative aspects of patients’ experience. If we had taken a role‐based approach we might have assumed that in these cases companions are acting intrusively (Adelman et al., 1987). Using established coding schemes might have led us to conclude that with those actions companions undermine patients’ autonomy (Clayman et al., 2005). Our analyses yielded a different picture. All participants in our data work to support patients’ right to speak for themselves. HCPs address questions and offers to patients and ostensibly wait for them to respond. Companions hold off intervening until patients have had an opportunity to do so (with only one exception—Extract 6—which we explained with reference to contextual circumstances). The design of companions’ actions further embodies their deference to patients’ prerogative to have the final say on matters pertaining to them—a prerogative that patients recurrently claim. In all these ways, companions’ interventions on patients’ behalf are collaboratively constructed as warranted departures from an otherwise prevailing normative orientation to patients’ *right to speak for themselves*. The practices we identified support and maintain this right and can thus be considered one fundamental way in which the principle of patient autonomy is put into practice within health‐care interactions.

Our study further contributes to the sociology of health and illness by characterising how companions’ actions contribute to shaping relationships within health‐care interactions. Our findings show that companions are not just accessories whose participation is peripheral relative to the axis of the HCP‐patient relationship. Rather, companions and patients work (and are treated) as relational units or ‘interactional teams’ (Lerner, 1993) in their interactions with HCPs. These teams share intimate knowledge about the patient's life, and companions actively help articulate it in specific moments. At the same time, patient‐companion teams have an internal organisation whereby patients hold primary rights to articulate their own experience—a right to which companions are bound to defer. These findings resonate with philosophical and ethical discussions, such as van Nistelrooij et al.’s (2017) analysis of patients’ autonomy in relational (rather than individualistic) terms through the recognition of family members’ contributions to patients’ decision‐making. This relational configuration appears to fit the palliative care setting in which we recorded our data. Companions who attend hospice consultations are involved in the care and support of adult patients who are older, frailer and manage long‐term and life‐limiting conditions. They are likely to act as invested parties who share significant knowledge about patients’ circumstances. Our study documents how this orientation is embodied in actions through which companions occasionally act as extensions of patients, sharing delicate aspects of patients’ experience on their behalf. However, it is reasonable to expect that the distributions of rights and responsibilities we have documented apply to other types of setting. Future studies should explore how patients’ and companions’ relative rights and responsibilities are managed in other settings and activities.

Companions further contribute to the organisation of health‐care interactions through interventions that assist and qualify patient‐HCP transactions. Companions can help forward activities initiated by an HCP, which may have temporarily stalled, such as when a patient does not answer a question (Extract 5) or when a patient observably hesitates to respond to an offer of services, thus projecting a possible rejection (Extract 4). Companions can also intercede on behalf of patients in circumstances where interactional tensions arise, such as when an HCP repeatedly seeks to overcome a patient's displayed reluctance to accept offers of services (Extract 6). In these cases, companions can be seen to operate at the intersection of HCP’s and patients’ interactional projects, helping forward the activities that HCPs initiate whilst concurrently promoting patients’ interests.^1^

### Implications for practice

Our findings have implications for how HCPs can support companions’ participation. A practical problem for companions is how to time their interventions on behalf of patients in ways that do not circumvent patients' primary right to speak for themselves. HCPs can open up opportunities for companions to intervene by asking them questions (Extract 2) and by gazing at them (Extract 3) at specific points in the interaction (importantly, after the patient has been given a first opportunity to speak for themself).

## CONCLUSION

Companions are invested parties who play an important part in the care and support of patients—especially when patients are older, frailer and/or manage long‐term and life‐limiting conditions. They are likely to possess a great deal of knowledge of patients’ condition and circumstances, and they may have observations or concerns they wish to raise within health‐care interactions. However, opportunities to do so are constrained by an overarching normative orientation to patients’ right to speak for themselves. This tension is arguably at its highest in the circumstances we have examined, which entail disclosing highly sensitive aspects of patients’ experience on their behalf. The practices we have documented are situated solutions that enable companions to perform circumscribed incursions into patients’ domain whilst concurrently displaying deference to their primary right to speak for themselves, ultimately preserving their autonomy.

## CONFLICT OF INTEREST

The authors have declared that no conflicts of interest exist.

## AUTHOR CONTRIBUTIONS

**Marco Pino:** Conceptualization (equal); Data curation (equal); Formal analysis (equal); Writing – original draft (lead); Writing – review & editing (equal). **Victoria Land:** Conceptualization (equal); Data curation (equal); Formal analysis (equal); Writing – review & editing (equal).

## ETHICAL APPROVAL

Ethics approval to collect the data was given by UK NRES Committees: Coventry & Warwickshire (Ref: 14/WM/0128) in 2014, and Nottingham 2 (Ref: 17/EM/0037) in 2017.

## PATIENT CONSENT

All participants gave consent for inclusion of pseudonymised transcripts in publications.

## Data Availability

The primary data for the study consist of a corpus of audio/video recorded health‐care consultations. The authors’ study protocols do not allow them to share the data beyond the research team in order to protect the participants’ confidentiality. However, the pseudonymised transcripts examined in this paper can be obtained upon request by contacting the first author.

## ENDNOTE

^1^
We do not mean to suggest that the companions in our data never take positions that are at odds or even conflict with those that the patients endorse. This happens in our data within other sequences of action than the ones we have examined in this report, and we have documented this elsewhere (Pino et al., 2021).

## Appendix

Transcription conventions adapted from Jefferson (2004):

**Table:**

,	Slightly upward intonation
¿	Upward intonation (more marked than a comma but less than a question mark)
?	Upward intonation
.	Falling intonation
_	Level intonation
[	Overlapping talk begins
]	Overlapping talk ends
(0.8)	Silences in tenths of a second
(.)	Silence less than two‐tenths of a second
wo:::rd	Lengthening of the sound just preceding
wo‐	Abrupt cut‐off or self‐interruption of the sound in progress
word	Stress or emphasis (usually conveyed through slightly rising intonation)
↑ ↓	Marked pitch rise or fall
=	Latching
( )	Talk too obscure to transcribe
(word)	Best estimate of what is being said
hhh	Hearable out‐breath
.hhh	Hearable in‐breath
w(h)ord	Aspiration internal to a word
((words))	Transcriber comments
°word°	Quieter or softer talk
°°word°°	Particularly quiet voice or whispering
WORD	Louder talk
>word<	Faster or rushed talk
<word>	Slower talk
£word£	Talk delivered with a smiley voice quality
#word#	Talk with a creaky voice quality

Conventions for the transcription of visible actions adapted from Mondada (2018):

**Table:**

% %	Descriptions of visible action are delimited between
+ +	two identical symbols (one symbol per participant's line of action) and are synchronised with corresponding stretches of talk/lapses of time
*‐‐‐>	The action described continues across subsequent lines
‐‐‐>*	until the same symbol is reached
>>	The action described begins before the extract's beginning
‐‐>>	The action described continues after the extract's end
........	Action preparation
‐‐‐‐‐‐	Full extension of the action is reached and maintained
p‐john	Participant doing the embodied action is indicated in lower case when they are not the speaker
